# *De Novo* Genome Assembly and Comparative Genomics of the Barley Leaf Rust Pathogen *Puccinia hordei* Identifies Candidates for Three Avirulence Genes

**DOI:** 10.1534/g3.119.400450

**Published:** 2019-08-23

**Authors:** Jiapeng Chen, Jingqin Wu, Peng Zhang, Chongmei Dong, Narayana M. Upadhyaya, Qian Zhou, Peter Dodds, Robert F. Park

**Affiliations:** * Plant Breeding Institute, School of Life and Environmental Sciences, Faculty of Science, The University of Sydney, Cobbitty, NSW, Australia; † Commonwealth Scientific and Industrial Research Organisation, Agriculture and Food, Canberra, ACT, Australia, and; ‡ Agricultural Genomic Institute at Shenzhen, Chinese Academy of Agricultural Sciences, Shenzhen 518124, China

**Keywords:** barley leaf rust, genome assembly, comparative genomics, gene-for-gene, avirulence gene, resequencing

## Abstract

*Puccinia hordei* (*Ph*) is a damaging pathogen of barley throughout the world. Despite its importance, almost nothing is known about the genomics of this pathogen, and a reference genome is lacking. In this study, the first reference genome was assembled for an Australian isolate of *Ph* (“*Ph*560”) using long-read SMRT sequencing. A total of 838 contigs were assembled, with a total size of 207 Mbp. This included both haplotype collapsed and separated regions, consistent with an estimated haploid genome size of about 150Mbp. An annotation pipeline that combined RNA-Seq of *Ph*-infected host tissues and homology to proteins from four other *Puccinia* species predicted 25,543 gene models of which 1,450 genes were classified as encoding secreted proteins based on the prediction of a signal peptide and no transmembrane domain. Genome resequencing using short-read technology was conducted for four additional Australian strains, *Ph*612, *Ph*626, *Ph*608 and *Ph*584, which are considered to be simple mutational derivatives of *Ph*560 with added virulence to one or two of three barley leaf rust resistance genes (*viz. Rph3*, *Rph13* and *Rph19*). To identify candidate genes for the corresponding avirulence genes *AvrRph3*, *AvrRph13* and *AvrRph19*, genetic variation in predicted secreted protein genes between the strains was correlated to the virulence profiles of each, identifying 35, 29 and 46 candidates for *AvrRph13*, *AvrRph3* and *AvrRph19*, respectively. The identification of these candidate genes provides a strong foundation for future efforts to isolate these three avirulence genes, investigate their biological properties, and develop new diagnostic tests for monitoring pathogen virulence.

Leaf rust, caused by the fungus *Puccinia hordei* (*Ph*), is a damaging disease of barley that has caused substantial yield losses as high as 62% (Cotterill *et al.* 1992; 1995). One of the most cost effective methods to manage this disease is the use of resistance genes (R genes) (Park 2008). Many of the R genes deployed in agriculture to control rust are thought to encode immunoreceptors that recognize avirulence (Avr) gene products, or effector proteins, from invading rust pathogens. This recognition leads to the initiation of the host immune response to arrest rust development around the infection site, often leading to a localized hypersensitive reaction (Jones and Dangl 2006; Dodds and Rathjen 2010). In this model, the evolution of new rust strains is thought to involve modification or deletion of Avr genes, which allows the pathogen to evade detection by the R gene product and infect a previously resistant variety. Identifying these Avr genes in the pathogen and the corresponding R genes in the host is an important step in understanding genetic interactions in the *Ph*-barley pathosystem and in developing new sustainable approaches and diagnostics to reduce the threat posed by this pathogen.

While the study of rust pathogen biology has been limited by their obligate biotrophic nature, Next-generation sequencing (NGS) technology has already resulted in the whole genome sequencing of several rust species and enabled significant advances in this field. To date, reference genomes have been assembled for four other cereal rust pathogens, namely *P. graminis* f. sp. *tritici* (*Pgt*), *P. triticina* (*Pt*), *P. coronata* f. sp. *avenae* (*Pca*), and *P. striiformis* f. sp. *tritici* (*Pst*) (Duplessis *et al.* 2011; Cuomo *et al.* 2017; Miller *et al.* 2018; Schwessinger *et al.* 2018). This has allowed initial cataloguing of genes that are believed to encode effector proteins that are secreted into the host and modulate the interaction between the pathogen and the host. Effector proteins are generally considered to contain a signal peptide for secretion, and no transmembrane domain (Saunders *et al.* 2012; Sperschneider *et al.* 2015; Sonah *et al.* 2016). The up-regulation of a given candidate effector gene in haustoria, the fungal structures considered to be the major site of effector secretion, is considered further evidence that it is likely to encode an effector protein (Dodds 2004; Garnica *et al.* 2014). A study of five Australian strains of *Pgt* identified 520 genes as candidate effectors based on their structure and up-regulation in haustoria compared to germinated urediniospores (Upadhyaya *et al.* 2015). In another comparative genomics study, non-synonymous nucleotide changes between two isolates of *Pst* led to the identification of five Avr candidates (Cantu *et al.* 2013). Similarly, comparative genomics and statistical association analysis found 20 candidates for the Avr gene corresponding to R gene *Lr20* in *Pt* (Wu *et al.* 2017).

With the availability of reference genomes, genome re-sequencing followed by variant calling has proven to be an effective method for Avr gene identification in plant pathogens. A typical workflow of re-sequencing analysis is to map sequencing reads from individual strains of different virulence profiles to a reference genome, and associate genotypic variations with virulence phenotypes based on mapping. For instance, re-sequencing of strains of the vascular wilt fungus *Verticillium dahliae* identified a 50 Kbp stretch of DNA present in four avirulent strains but absent in seven virulent strains, which led to the successful cloning of the Avr gene *Ave1* (de Jonge *et al.* 2012). In another study, comparative genomics of two strains of *Cladosporium fulvum* (causal agent of tomato leaf mold) differing in virulence to resistance gene *Cf-5* identified one gene with a 2 bp deletion, and confirmed that the gene was the corresponding Avr gene *Avr5* (Mesarich *et al.* 2014). More recently, comparison of the genomes of mutant strains of the wheat stem rust pathogen with acquired virulence for the resistance genes *Sr50* or *Sr35* identified the matching avirulence genes *AvrSr50* and *AvrSr35* (Chen *et al.* 2017; Salcedo *et al.* 2017). Taken together, these studies have demonstrated that the evolution of new virulence can be caused by non-synonymous mutations in Avr genes.

In Australia, although sexual reproduction of *Ph* has been observed (Wallwork *et al.* 1992), monitoring of strain (“pathotype”) evolution has suggested that the asexual cycle is dominant (Park 2003). One asexual (clonal) lineage within the pathogen population is thought to be derived from strain 5453P- (*Ph*560; Figure 1). This progenitor strain was first discovered in Western Australia in 2001, being pathogenically distinct from all *Ph* strains detected previously in Australia (Park *et al.* 2015). A derivative of this lineage that had acquired virulence to *Rph19* was detected in 2003 (strain 5453P+, *Ph*584) (Park *et al.* 2015). Later in 2008 and 2009, two further strains with independent virulence gains to *Rph13* and *Rph3* were detected (strains 5453P+ +Rph13 (*Ph*608) and 5457P+ (*Ph*612), respectively (Karaoglu and Park 2013). Later, an isolate of strain 5457P- (*Ph*626) was detected in 2013, which likely arose from the progenitor strain 5453P- by gaining virulence to *Rph3* (Figure 1). Based on the gene-for-gene hypothesis (Flor 1971), it is assumed that one dominant Avr gene corresponds to each of the three resistance genes *Rph3*, *Rph13* and *Rph19*, *viz*. *AvrRph3*, *AvrRph13* and *AvrRph19*, respectively and that virulence evolution in this lineage resulted from single independent mutation events in these genes.

**Figure 1:**
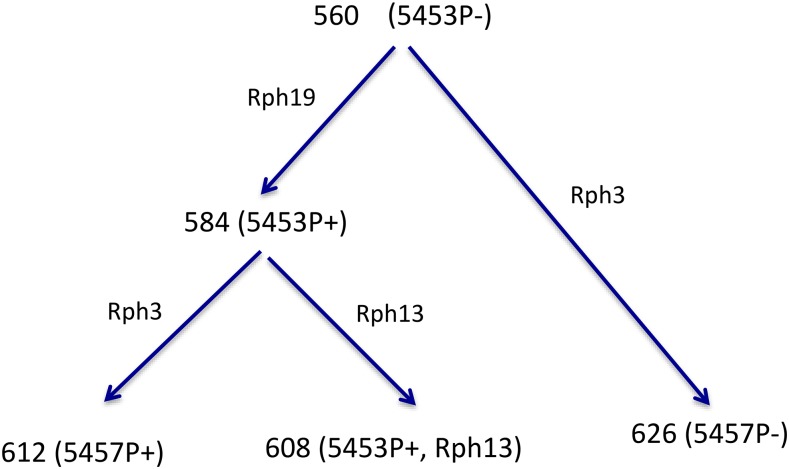
Proposed evolutionary pathways from the 5453P- lineage of *Puccinia hordei* based on pathogenicity testing. Nodes in the tree show culture numbers and strains in bracket, and the labels on tree branches show resistance genes overcome by the mutation.

In this study, whole genome sequencing was performed for five isolates of strains within the 5453P- lineage. The genome sequence of strain *Ph*560 was assembled from long read SMRT sequencing data to generate the first reference assembly for this pathogen species. A total of 25,543 gene models were predicted and those encoding secreted proteins (SPs) were identified. Re-sequencing data of isolates of the five strains were mapped to the reference genome to examine genetic variations that may account for their pathogenic differences on resistance genes *Rph3*, *Rph13* and *Rph19*.

## Materials and Methods

### DNA preparation and genome sequencing

The five strains used in this study were identified in annual Australia-wide national surveys of pathogenicity in *P. hordei* (R.F. Park, unpublished data), and are curated in the Plant Breeding Institute Rust Collection, The University of Sydney, Australia. Each isolate was established from a single pustule on an infected barley leaf to ensure purity. For PacBio sequencing of *Ph*560, a modified CTAB DNA extraction procedure (Schwessinger 2016) was used. The DNA solution obtained was then further purified to separate high molecular weight DNA from other impurities and low molecular weight DNA as described by Dong (2017). DNA concentration and purity was measured with a Qubit 3 (Invitrogen) and Nanodrop ND-1000 (Thermo Fisher Scientific). If the reading from the Qubit to Nanodrop ratio was smaller than 0.5, AMPure beads (Beckman, Coulter Inc.) were used to purify the DNA at the ratio of 0.45 beads to DNA (vol/vol) following the manufacturer’s protocol. DNA integrity was checked by pulsed-field gel electrophoresis. The sequencing library was prepared using SMRT cell Template Prep Kit 1.0-SPv3 with BluePippin Size-selection with 15-20 kb cutoff, and then sequenced using Sequel Sequencing Kit 2.0 at the Ramaciotti Centre for Genomics (Sydney, Australia).

For Illumina sequencing of the five strains, the CTAB extraction method (Rogers *et al.* 1989) was used to prepare DNA samples. For the genomic sequencing of *Ph*612, a library with insert size of 500 bp was sent to BGI Tech Solutions (Hong Kong) Co., Ltd. and was sequenced 90 bp paired-end on Illumina HiSeq 2000 platform. DNA extracted from *Ph*560 was sent to Novogene (HK) Ltd. for sequencing on Illumina HiSeq 2500 platform (PE 125bp), and the other three strains (*i.e.*, *Ph*626, *Ph*584 and *Ph*608) were sent to the Australian Genome Research Facility (AGRF) for sequencing on Illumina HiSeq 2500 (PE 125bp).

### Genome assembly

The raw PacBio sequencing reads of *Ph*560 were first processed using the read correction module of Canu v1.6 (Koren *et al.* 2017) with “genomeSize=220m” and the corrected reads were then assembled using SMARTdenovo version a411def (Ruan 2015) with default parameters. To polish the assembly, Illumina reads were mapped to the contigs with BWA mem 0.7.5a-r405 (Li 2013) and the resulting mapping file was subjected to Pilon 1.22 (Walker *et al.* 2014) to fix bases, fill gaps and correct local mis-assemblies. This mapping and fixing pipeline was run for three iterations.

### Read mapping, variant calling and annotation

Illumina reads of the five strains were trimmed with Trim Galore v0.3.7 (Martin 2011) with parameters “-quality 20 -phred33 -length 35”, and then mapped to Gn560 with BWA mem 0.7.5a-r405 (Li 2013). To minimize false positives of SNP calls around insertion/deletion (InDel) regions, poorly aligned reads around InDel were identified and realigned locally using RealignerTargetCreator and IndelRealigner in GATK package 3.8.1 (McKenna *et al.* 2010). The subsequent mapping files were used to call SNPs and InDels using GATK HaplotypeCaller (McKenna *et al.* 2010). The identified SNPs and InDels were annotated with SnpEff v4.1 (Cingolani *et al.* 2012) using default parameters. To manually curate the SNP calls produced by GATK, reads were mapped to the reference genome using Bowtie2 v2.2.5 (Langmead and Salzberg 2012) using parameters “-k 2–very-sensitive”. The parameter “-k 2” was selected to allow up to two mapping for a single read. The resulting read alignments were inspected using IGV to confirm the GATK SNP calls.

### Phylogenetic analysis

The evolutionary relationships between *Ph*560 and the four putative derivative strains identified in annual pathogenicity surveys (*Ph*584, *Ph*608, *Ph*612 and *Ph*626) were examined based on genome wide SNPs inferred using Poppr, an R package for population genetics analysis. Genetic distance was calculated using bitwise.dist function and 1,000 bootstrap replicates were performed.

### RNA isolation and sequencing

Barley seedlings (cultivar Morex) were infected with *Ph* strain 612. Infected leaves were harvested at 4 and 7 days post inoculation (dpi) and stored at -80 ◦C. Total RNA was extracted from infected leaf tissue using TRIzol reagent (Life Technologies Australia Pty Ltd) according to the manufacturer’s instructions. The total RNA was then treated with RNase-free DNase I (New England BioLabs Inc.), and column purified using ISOLATE II RNA Mini Kit (Bioline Australia) according to the manufacturer’s instruction. The quantity and quality of the total RNA were examined by Nanodrop (Thermo Scientific) and Agilent 2100 Bioanalyzer (Agilent Technologies). Two micrograms of RNA from 4 dpi and 7 dpi were combined. Library was constructed using a TruSeq Stranded mRNA-Seq Library Prep kit and was sequenced on HiSeq 2000 (PE 100bp) at Ramaciotti Centre for Genomics.

### Gene prediction

The raw RNA-Seq reads were trimmed with Trim Galore v0.3.7 (Martin 2011) with parameters “-quality 20 -phred33 -length 35”. The trimmed reads were aligned to Gn560 using TopHat v2.0.14 (Kim *et al.* 2013) with parameter setting “min-intron-length 10 -max-intron-length 5000 -mate-inner-dist 100 -mate-std-dev 100 -min-segment-intron 10 -max-segment-intron 5000”. Transcripts were assembled based on the mapping using Cufflinks v2.2.1 (Trapnell *et al.* 2010) with parameters “min-intron-length 10 -max-intron-length 5000 -minisoform-fraction 0.1”. To complement the RNA-Seq based prediction, protein sequences from *Pca*, *Pst* and *Pt* (Cuomo *et al.* 2017; Miller *et al.* 2018;) were mapped to Gn560 with MAKER v2.31.8 (Holt and Yandell 2011). Both transcript and protein mapping on Gn560 were used to predict gene structures with the MAKER pipeline.

### Functional annotation

The putative protein sequences were searched against the non-redundant protein database v2016-02-29 and SwissProt database v2017-03-05 using BLASTP (Altschul *et al.* 1997) with parameters “-e 1e-5 -F F -a 4”. The BLASTP hits were filtered requiring a minimum 40% of the *Ph* query proteins matched target proteins and the match identities were not less than 30%. SignalP v4.1 (Petersen *et al.* 2011) was used to predict signal peptide with parameter “-c 140” that examine the first 140 aa in given protein sequences. TMHMM v2.0c (Sonnhammer *et al.* 1998) was used to predict transmembrane domains in all the resulting proteins with putative signal peptides. If the predicted transmembrane domain in a given protein did not overlap with its signal peptide, it was considered as a transmembrane protein and removed from the set of effector candidates.

### Data availability

All raw sequencing data have been deposited in NCBI SRA under the BioProject ID PRJNA495764. The genome assembly has been deposited at GenBank under the accession RDRW00000000, and consists of sequences RDRW01000001-RDRW01000838. Gene structure prediction is recorded in a gff format file available at https://github.com/chjp/Phordei.git. Supplemental material available at FigShare: https://doi.org/10.25387/g3.7823987.

## Results

### *De novo* assembly

The founder strain *Ph*560 was selected to assemble a reference genome for *P. hordei*. A total of 18.5 Gbp of PacBio SMRT sequencing data (Table 1) were generated for this strain. We used Canu to pre-correct the original reads and assembled the resulting data using SMARTdenovo. The resulting assembly was polished using three rounds of Pilon with 11.6 Gbp of 125bp paired-end sequencing data from the Illumina platform. The final assembly contained a total of 838 contigs with a size of 207 Mbp (Table 2). This set of contigs is referred to as Gn560 henceforth. The alignment rate of Illumina reads of *Ph*560 to this assembly was 95.4%, indicating a high level of genome completeness. To examine gene space completeness in the assembly, BUSCO (Simão *et al.* 2015) was used to map 1,335 highly conserved single-copy orthologs in the Basidiomycota lineage to the assembly. This analysis showed 93.5% (1,249/1,335) of the conserved gene set was present in Gn560.

**Table 1:** Sequencing information for five strains of *Puccinia hordei*

Strain	Library	Read Length (bp)	Insert Size (bp)	Data Size (bp)
*Ph*560	High molecular weight DNA	22,250 (N50)	20k	18.5G
*Ph*560	Paired-end DNA	125	350	11.6G
*Ph*626	Paired-end DNA	125	550	11.1G
*Ph*584	Paired-end DNA	125	550	11.8G
*Ph*608	Paired-end DNA	125	550	11.0G
*Ph612*	Paired-end DNA	90	500	42.0G
*Ph612*	RNA-Seq	101	330	50G

**Table 2:** *De novo* assembly statistics for *Puccinia hordei* strain *Ph*560

	Contig
Total Number (#)	838
Total Length (bp)	206,919,034
N50 (bp)	405,324
N90 (bp)	109,507
Min Length (bp)	20,948
Ave Length (bp)	246,920
Max Length (bp)	2,083,918
No. Sequence > 1 Mbp	40
Gap region (bp)	0
GC Content (%)	41.65
Complete and single copy BUSCOs	867
Complete and duplicated BUSCOS	382
Fragmented BUSCOs	50
Missing BUSCOs	36

**Figure 2:**
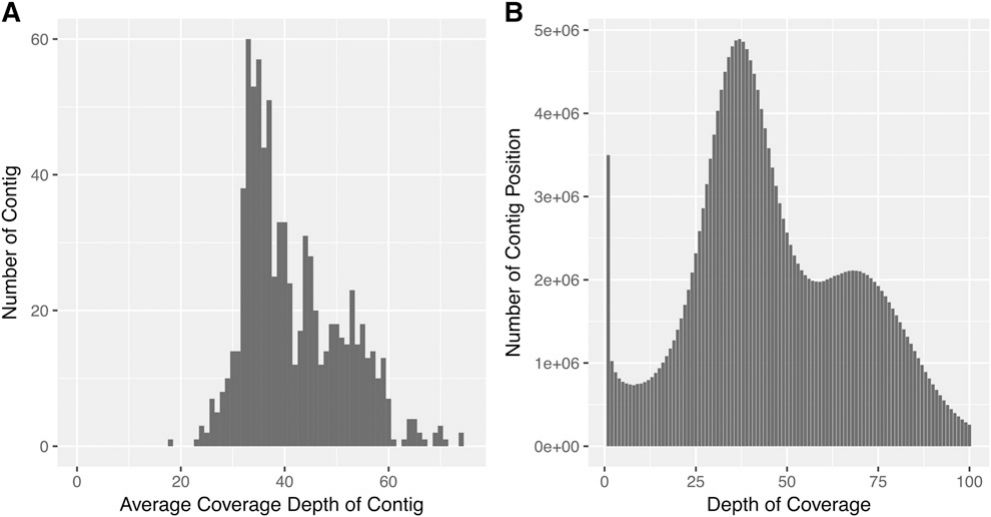
Coverage depth analysis of Illumina reads of *Ph*560 mapped back to the genome assembly. (A) Average coverage depth of contigs was calculated and the Y axis shows the number of contigs with a specific depth. Contigs with read depth higher than 75 are not shown. (B) Histogram of coverage depth over individual positions in all contigs.

Eilam *et al.* (1994) used flow cytometry to estimate relative genome sizes of *Pgt* and *Pca* as 56% and 64% of that of *Ph*. This is consistent with their haploid genome assembly sizes of 88 Mbp (Duplessis *et al.* 2011) and 100 Mbp (Miller *et al.* 2018), respectively, and gives an estimated haploid genome size of 156 Mbp for *Ph*. We suspected that the larger assembly size was caused by high heterozygosity in some genomic regions preventing haploid assembly, resulting in assembly of two copies representing the two haplotypes of the uredinial stage of this organism. This suspicion was supported by Illumina read coverage analysis of the assembly, which showed a two-peak distribution of depth for both whole contigs and individual bases (Figure 2; Supplementary Table S1). This is expected when mixed haplo-separated and collapsed regions are present within the assembly (Miller *et al.* 2018), as collapsed regions will show twice the coverage of haplo-separated regions because reads from both alleles will map to the same position. Thus the first peak at ∼37X would represent the read depth at haplo-separated positions, while the second peak of ∼72X indicates the merged haploid assembly. In addition, 382 out of 1,249 (31%) complete genes identified in the BUSCO analysis were present in two copies (Table 2), probably due to the allele separation on highly heterozygous regions. These numbers suggest that about two thirds of the haploid genome content of our assembly is represented in single copy collapsed sequences, while the remaining third is represented by duplicated contigs. This suggests a haploid genome size of about 150Mbp, consistent with the flow cytometry estimates relative to *Pgt* and *Pca*.

### Gene prediction

Under optimal conditions, *P. hordei* has colonized its host and produced infection structures by 2 days after infection, while the process of spore production commences within 6-8 days of infection (Clifford 1985). To guide gene annotation, RNA-Seq was therefore performed for infected barley leaf tissues collected at two time points (the 4^th^ and 7^th^ day) post inoculation with *Ph*612. The RNA-Seq reads were aligned to the draft genome Gn560 with TopHat2. A total of 38.6% of the reads were successfully aligned and retained, whereas the remaining 61.4% of reads were discarded as derived from the barley leaf transcriptome. The read alignment was then assembled to transcripts using Cufflink. These transcripts should capture a majority of the genes expressed *in planta* during active colonization and sporulation.

To obtain a comprehensive gene repertoire, timepoints representing all life stages should be covered. Therefore, proteins predicted at more life stages than those sampled here in three other *Puccinia* species (*viz*. *Pt*, *Pca* and *Pst*) were used to complement the RNA-Seq data. These protein sets include genes expressed in dormant urediniospores, germinating urediniospores, isolated haustoria and during host infection. Both the *P. hordei* transcripts and the *Puccinia spp*. proteins were mapped to Gn560 using the MAKER pipeline, resulting in the prediction of 25,543 genes and 66,126 isoforms. The translated protein sequences from the gene isoforms were aligned to NCBI nr and Swissprot databases for functional annotation (Supplementary Table S2). The predicted genes were named with a common prefix “PH560” indicating their origin from *P. hordei* strain *Ph*560, and a unique suffix number derived from the prediction tool MAKER (*e.g.*, *PH560_12290*).

### Effector prediction

Rust fungi secrete effector proteins into the plant apoplast or cytoplasm and secretome prediction based on the presence of secretion signal peptides at the N termini of proteins can be used to identify candidate effectors. A scan of the *Ph* proteome with SignalP4.1 identified 1,739 genes encoding a signal peptide (Supplementary Table S3). Of these, 289 were excluded because they contained a non-overlapping predicted transmembrane segment that is associated with membrane integration, leaving a total of 1,450 candidate genes predicted to encode secreted proteins.

MAKER mapped the protein sequences and RNA-Seq reads to the genome and predicted gene structures by integrating evidence from both. With a cutoff of mapping coverage 90%: 121 SP genes were supported by RNAseq but not by protein mapping; 675 SP candidates were covered with <90% by RNAseq but more than 90% by proteins; and the remaining 654 SP candidates were supported by both sources in integration.

### Read mapping

In order to identify candidates for the three Avr genes in which mutations had occurred within the *Ph*560-derived lineage, we generated Illumina short read genomic DNA sequencing data for the four mutation-derived strains (*Ph*612, *Ph*608, *Ph*584, and *Ph*626) within this lineage. This yielded at least 11 Gbp (∼70X average coverage) data for each strain after quality trimming (Table 1). To genotype the strains, the sequencing reads of each isolate were mapped to the reference genome Gn560 individually. For each strain, about 95% of reads could be mapped to the reference and the percentage of the reference coverage was over 99%, suggesting that almost all genes annotated in the reference could be genotyped.

### Phylogenetic analysis

**Figure 3:**
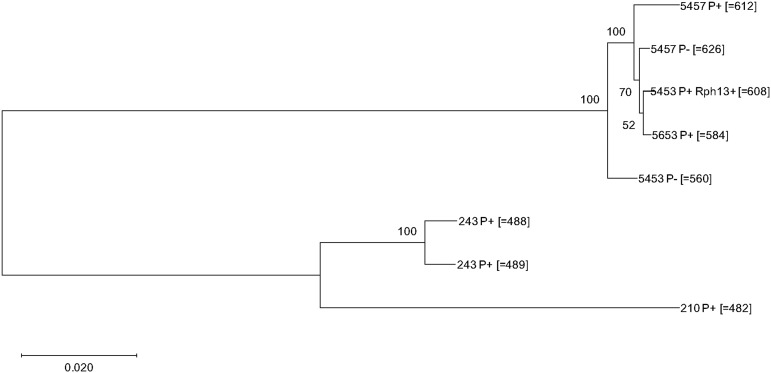
Dendrogram of eight *Puccinia hordei* strains based on the total identified SNPs inferred using Poppr. Genetic distance was calculated using bitwise.dist function. The numbers shown on the dendrogram branches are the percentage of bootstrap replicates (1,000) supporting the cluster.

To confirm the relatedness of the strains within the putative clonal lineage founded by strain 5453P, a phylogenetic tree including *Ph*560, the four presumed derivative strains, and three pathogenically distinct *P. hordei* strains collected in 1966 (*Ph*488, *Ph*489) and 1990 (*Ph*482) was constructed based on the genome-wide SNPs identified (Figure 3). While the three distant strains and *Ph*560 derived series formed two distinct clusters, within the *Ph*560 cluster, *Ph*584 and *Ph*608 formed a sister group and *Ph*626 and *Ph*612 formed distinct branches. The overall topology of the phylogenetic tree was consistent with the hypothesis that strains *Ph*584, *Ph*608, *Ph*612 and *Ph*626 were derived from *Ph*560 via simple mutational acquisition of virulence for the resistance genes *Rph3*, *Rph13*, and *Rph19*.

### Genome wide polymorphism

To compare genotypes across the five strains, small variations including single nucleotide polymorphisms (SNPs) and insertion/deletions (InDels) between individual strains were examined based on mapping reads to the reference using GATK HaplotypeCaller (McKenna *et al.* 2010). For the putative progenitor strain *Ph*560, 516,533 variants were identified, occurring at a genome-wide frequency of 2.5/Kbp with over 90% present in a heterozygous state. Among the variants, SNPs and InDels showed a ratio of 4.8:1. The total number of sequence variants identified for the remaining four strains ranged from 552,081 to 554,627 and showed similar ratios of SNP/InDel (Table 3).

**Table 3:** Genomic variant calling in five *Puccinia hordei* strains

Strain	Mapping rate	Total variants	SNP	Insertion	Deletion	Homozygous	Heterozygous
*Ph*560	95.37	516,533	427,589	44,700	44,244	31,809	484,724
*Ph*626	95.04	554,627	452,714	51,072	50,841	43,617	511,010
*Ph*584	95.01	554,012	451,548	51,359	51,105	43,185	510,827
*Ph*608	94.31	552,573	450,860	50,875	50,838	43,416	509,157
*Ph*612	85.46	552,081	453,892	49,924	48,265	41,602	510,479

In order to relate genomic variants to gene structure, SnpEff (Cingolani *et al.* 2012) was used to map all SNPs and InDels to exons and splice sites to evaluate their impact on protein coding (Table 4). An InDel in a coding region can cause a reading frame shift if its size is not a multiple of three, whereas an InDel of one or several codons induces a less severe effect on protein coding. InDels of these two types were referred to as frameshift and inframe InDels, respectively. The average counts of frameshift and inframe mutations in the five strains were 5,409 and 1,339, respectively. In addition to InDels, sequence changes in the start and stop codons may also have a high impact on gene function, as loss of a start codon would abolish protein translation, gain of a stop codon would cause premature protein truncation, and a stop codon loss would result in additional amino acids (aa) that may change protein structure. These three types of mutations occurred on average 191, 2,629 and 779 times, respectively, in the five strains. Furthermore, aa changes caused by nonsynonymous (NSY) SNPs may also have direct functional implications, whereas synonymous (SYN) SNPs resulting in no aa change may not have such an effect. Across the five strains, more NSY variants were detected as compared to the SYN variants, and the average counts of these two types were 67,526 and 46,763, respectively.

**Table 4:** Coding impacts of genomic variants for five strains of *Puccinia hordei*

Type	*Ph*560	*Ph*626	*Ph*584	*Ph*608	*Ph*612
Frameshift InDel	4,649	5,236	5,213	5,194	4,953
Inframe InDel	1,251	1,385	1,366	1,375	1,320
Start codon lost	183	203	195	197	178
Stop codon gained	2,517	2,693	2,674	2,695	2,568
Stop codon lost	714	835	802	830	714
Splice variant	320	344	335	346	396
Non-synonymous variant	64,173	68,938	68,636	68,585	67,297
Synonymous variant	44,805	47,489	47,278	47,570	46,672

### Genomic polymorphism in SP genes associated with avirulence/virulence

As effector proteins are most likely encoded by SP genes, our search for the three Avr genes *AvrRph3*, *AvrRph13*, and *AvrRph19* focused on the detection of mutations in candidate SP genes between the five *Ph* strains. A total of 714 SP genes contained protein coding polymorphisms in at least one of the five strains. The variants in these 714 genes were scanned manually for read count support in the read mapping to identify false calling. We found that 24 of these genes appeared to be multi-copy genes that had been collapsed into one locus in the assembly, because they contained sites with more than two alternative alleles and allele frequencies that diverged from the expected 1:1, and hence we were not able to genotype these genes. For the remainder, after manual curation, we confirmed the presence of 976, 1,023, 1,019, 1,240 and 1,023 polymorphic sites in SP genes of strains *Ph*560, *Ph*584, *Ph*608, *Ph*612 and *Ph*626, respectively (Supplementary Table S4). To identify candidates for the *AvrRph3*, *AvrRph13* and *AvrRph19* genes, we looked for amino-acid changing polymorphisms that were shared by isolates of the same virulence phenotype and that could therefore represent mutations determining these virulence changes in this lineage of *Ph*.

To identify candidates for *AvrRph13*, we compared the variant calls for the SP genes between strains *Ph*584 and *Ph*608. The two strains displayed different genotypes at 41 positions in 35 SP genes (Supplementary Table S5) in either a homozygous or heterozygous condition.

**Figure 4:**
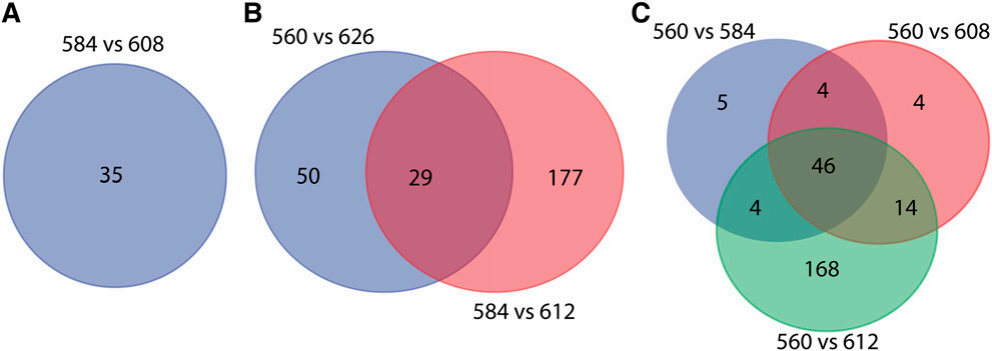
Venn diagrams for intersection and complement of genes encoding secreted proteins with non-synonymous mutations. (A), (B) and (C) demonstrate the candidates for *Rph13* (35), *Rph3* (29) and *Rph19* (46), respectively.

Based on pathogenicity plus geographic region and time of isolation, strains *Ph*626 and *Ph*612 are considered to have gained virulence to *Rph3* via independent single step mutations from their progenitor strains, *Ph*560 and *Ph*584, respectively. Therefore, comparisons between *Rph3*-virulent and avirulent strains were carried out, *i.e.*, *Ph*626 *vs.* *Ph*560 and *Ph*612 *vs.* *Ph*584, to detect the SP genes that showed functional variations. Because it was not known whether the gene had the same mutation for the two independent virulence gains, SP genes that showed protein polymorphism in both comparisons were considered as potential candidates. Within the SP genes, strains *Ph*560 and *Ph*626 showed differences in 123 sites distributed in 79 SP genes (Supplementary Table S5). Similar screening was performed to compare strains *Ph*584 and *Ph*612, which enabled the identification of 206 SP genes showing changes in protein coding ability. The gene sets resulting from the two comparisons shared a common panel of 29 genes (Figure 4B and Supplementary Table S5).

The strains *Ph*584, *Ph*612 and *Ph*608 were virulent for *Rph19* and were derived sequentially from the progenitor *Ph*560. The snpEff annotation indicated that 59, 70 and 232 SP genes displayed protein polymorphisms when comparing *Ph*560 with *Ph*584, *Ph*560 with *Ph*608, and *Ph*560 with *Ph*612, respectively. As *AvrRph19* had to differ in all three comparisons, the three sets of SP genes that showed protein variations were compared and 46 genes in common were thus identified (Figure 4C). According to the putative genetic relationship of *Ph*584, *Ph*612 and *Ph*608, the mutations causing the *AvrRph19* loss in *Ph*584 should be retained in its two derivatives *Ph*612 and *Ph*608. These new mutations should also be specific to *Ph*584, *Ph*612 and *Ph*608, and absent in *Ph*626. There were eight such mutations distributed in seven genes that are therefore considered the most promising candidates for *AvrRph19* (Supplementary Table S5).

## Discussion

This study reports the first genome assembly of the economically important cereal rust pathogen *P. hordei*, providing a valuable genomic resource for the rust research community. As a high-quality reference genome, the assembly enables genomic and transcriptomic comparisons of isolates within and across different *P. hordei* lineages (Park *et al.* 2015), especially in studies targeting diversity in candidate effector genes. Genomes of the currently sequenced cereal rust fungi differ significantly in size, ranging from 83 Mbp in *Pst* to 135 Mbp in *Pt* (Duplessis *et al.* 2011; Cuomo *et al.* 2017; Miller *et al.* 2018; Schwessinger *et al.* 2018). A previous flow cytometry study estimated the *Ph* genome size to be 122 Mbp (Kullman *et al.* 2005). The larger size of assembly of 207 Mbp reported here may have resulted from high heterozygosity in some genomic regions between the two nuclei that have prevented haploid assembly and resulted in two separate allelic contig sequences (Figure 2). Based on read coverage and the proportion of single copy and duplicated conserved genes, we estimated a total haploid genome size of about 150Mbp. The N50 of the *Ph* contigs generated in the present study is 405,324 bp, comparable to two recently published genome assemblies for rust species based on PacBio sequencing (Miller *et al.* 2018; Schwessinger *et al.* 2018).

Transcriptome sequencing of infected leaf tissues collected 4 and 7 days post inoculation was used to guide the prediction of *Ph* genes expressed in hyphae and haustoria at these two time points. In addition, the alignment of *Pgt*, *Pca*, *Pt* and *Pst* proteins to the *Ph* genome enabled the identification of conserved genes missing in the *Ph* RNA-Seq data due to their exclusive expression at other life stages or in other tissues. The combination of these two types of evidence allowed prediction of a comprehensive gene space for the damaging uredinial stage of *Ph* on barley. To build on the gene annotation work reported here, future efforts will focus more on biological functions, such as identifying secreted protein-encoding genes preferentially expressed in haustoria, the presumed sites for effector delivery into host shown to be enriched for effector genes in *Melampsora lini* (Catanzariti *et al.* 2006).

To identify candidate Avr genes in *Ph*, comparisons were made between the whole genome sequences of isolates of five *Ph* strains that comprised a progenitor and four presumed mutational derivatives (Figure 1) differing in virulence for three barley leaf rust resistance genes, *viz. Rph3*, *Rph13* and *Rph19*. The sequencing reads of the five strains were mapped to the reference genome Gn560 generated for the presumed progenitor *Ph*560. The mapping rates ranged from 85–95% with an average rate of 93%, similar to rates observed for Illumina mapping to the highly contiguous PacBio assemblies of *Pca* (Miller *et al.* 2018) and *Pst* (Schwessinger *et al.* 2018), and higher than observed for short read or Sanger based assemblies of other rust fungi (Duplessis *et al.* 2011; Cantu *et al.* 2013; Nemri *et al.* 2014; Upadhyaya *et al.* 2015 Cuomo *et al.* 2017). A phylogeny based on whole genome SNPs supported the clonal derivation of the five strains, indicating that the virulence differences between them should be the result of mutation.

It is not known whether the progenitor strain *Ph*560 is homozygous or heterozygous at the three Avr loci *AvrRph3*, *AvrRph13* and *AvrRph19*. If homozygous, both alleles of each Avr gene would need to mutate in order for virulence gain; if heterozygous, mutation of the single Avr allele only is required. In this study, both homozygous and heterozygous polymorphisms in the virulent strains were analyzed. By focusing on 1,450 SP genes, this study identified 29, 35 and 46 genes that encode proteins polymorphic between strains with differential virulence profiles for the R genes *Rph3*, *Rph13* and *Rph19*, respectively (Figure 4) and seven genes containing common mutations in all three *Rph19* virulent strains. Given that the resistance genes *Rph3*, *Rph13*, and *Rph19* have not yet been isolated from barley, functional testing of the candidate Avr genes will require either an *in-planta* expression systems to express the genes in barley lines containing *Rph3*, *Rph13*, and *Rph19*, or testing in a heterologous system such as a viral overexpression system (Lee *et al.* 2012) or a protoplast expression assay (Lu *et al.* 2016). The functional study of these candidate genes will provide deeper understanding of the *Ph*-barley pathosystem.

Compared with other cereal rust species present in Australia, *Ph* is the only one that undergoes sexual recombination (Wallwork *et al.* 1992), providing an opportunity to use a map-based approach to help isolate Avr genes including those targeted in the current study. Sexual crosses of *Ph* isolates would permit assessments of co-segregation between the candidates identified in the present study and virulence among F_2_ progeny. This research method has provided substantial knowledge of the flax rust-flax interaction, leading to the isolation of several Avr genes (Dodds 2004; Catanzariti *et al.* 2006; Anderson *et al.* 2016). Apart from traditional map-based cloning, genome wide association studies (GWAS) by sequencing could also serve as a powerful tool for Avr gene identification in isolates of *Ph* derived from sexual recombination. This has been shown to be efficient in several studies of plant pathogen populations with sexual recombination (Lu *et al.* 2016; Plissonneau *et al.* 2017; Praz *et al.* 2017).

The whole genome sequencing data of five strains reported here provides a valuable resource for developing new DNA markers that will be useful for genetic studies and population monitoring of the *Ph* 5453P- lineage in Australia and beyond. Diagnostic simple sequence repeat (Du *et al.* 2012; Kitchen *et al.* 2012; Karaoglu and Park 2013; Metz *et al.* 2016) and single nucleotide polymorphism (SNP) markers have been and will be further developed for *P. hordei* based on genome re-sequencing data. Combined with these tools, the genome sequence data should prove very useful in contributing to the development of more sustainable rust control, surveillance and management strategies for this important barley pathogen.
